# Local Extremum Mapping for Weak Supervision Learning on Mammogram Classification and Localization

**DOI:** 10.3390/bioengineering12040325

**Published:** 2025-03-21

**Authors:** Minjuan Zhu, Lei Zhang, Lituan Wang, Zizhou Wang, Yan Wang, Guangwu Qian

**Affiliations:** 1 College of Computer Science, Sichuan University, Section 4, Southern 1st Ring Rd., Chengdu 610065, China; zhuminjuan@stu.scu.edu.cn (M.Z.); leizhang@scu.edu.cn (L.Z.); lituanwang@scu.edu.cn (L.W.); 2 Institute of High Performance Computing, Agency for Science, Technology and Research (A*STAR), Singapore 138632, Singapore; wang_zizhou@ihpc.a-star.edu.sg (Z.W.); wangyan@ihpc.a-star.edu.sg (Y.W.); 3 West China Biomedical Big Data Center, West China Hospital, Sichuan University, Section 4, Southern 1st Ring Rd., Chengdu 610065, China

**Keywords:** mammography images, breast cancer classification, lesion localization, weak supervision, deep neural networks

## Abstract

The early and accurate detection of breast lesions through mammography is crucial for improving survival rates. However, the existing deep learning-based methods often rely on costly pixel-level annotations, limiting their scalability in real-world applications. To address this issue, a novel local extremum mapping (LEM) mechanism is proposed for mammogram classification and weakly supervised lesion localization. The proposed method first divides the input mammogram into multiple regions and generates score maps through convolutional neural networks. Then, it identifies the most informative regions by filtering local extrema in the score maps and aggregating their scores for final classification. This strategy enables lesion localization with only image-level labels, significantly reducing annotation costs. Experiments on two public mammography datasets, CBIS-DDSM and INbreast, demonstrate that the proposed method achieves competitive performance. On the INbreast dataset, LEM improves classification accuracy to 96.3% with an AUC of 0.976. Furthermore, the proposed method effectively localizes lesions with a dice similarity coefficient of 0.37, outperforming Grad-CAM and other baseline approaches. These results highlight the practical significance and potential clinical applications of our approach, making automated mammogram analysis more accessible and efficient.

## 1. Introduction

According to the latest cancer statistics [1], breast cancer has been the most common cancer among women, alone accounting for 31% of female cancers. The research of the World Health Organization has shown that early detection can have a positive impact on the prognosis and survival rate of breast cancer.

Mammography is widely recognized as an effective way to reduce mortality, and it has become a common tool in the early detection of breast cancer [2]. To help radiologists improve the mammography screening process and reduce their workloads, many computer-aided diagnosis (CAD) systems based on mammography images have been proposed to identify benign and malignant breast tumors [3,4,5,6]. With the surge of interest in deep neural networks (DNNs), it has been widely used in automatic mammography image analysis and has achieved great success in CAD systems. Existing DNN-based methods can generally be divided into the following two types.

The first treats breast cancer screening as a detection or segmentation task [7,8,9], focusing on locating or outlining lesions in mammograms and then classifying them as benign or malignant. These types of models can provide detailed lesion information and auxiliary evidence for radiologists to diagnose a mammographic image. Typically, sophisticated annotations are required for their construction, e.g., bounding boxes or pixel-level segmentation ground truth. These annotations are usually made by experienced radiologists to ensure accuracy, which requires a lot of effort and high costs. So, it is difficult to acquire a large amount of annotated data to build this type of model. For the same reason, a large number of unprocessed mammograms in hospitals have not been utilized due to a lack of pixel-level annotations. However, their image-level annotations can be easily queried from pathology reports. Therefore, how to reasonably utilize this part of the data to improve the efficiency of breast cancer screening has become an important question.

The second type of method focuses on establishing classification frameworks with only image-level annotations to predict the existence or absence of benign and malignant lesions in mammograms [10,11,12]. This method analyzes a large number of mammograms without detailed pixel-level annotations and fully mines its underlying information. However, due to the limitation of annotations, these models often fail to provide detailed location information. This information is very important because it can help radiologists quickly locate the support regions of model predictions. The absence of this information means that radiologists need to manually look for suspicious locations to verify the accuracy of predictions. Therefore, it is necessary to construct a method that can provide location information with only image-level annotation. To address this, several weakly supervised algorithms have been proposed that address the challenge of classifying mammograms and locating lesions with only image-level annotations [13,14,15,16]. However, they have usually provided only the location of the malignant lesions or introduce additional computational overhead.

In this study, a weakly supervised algorithm is proposed to address the challenges of classifying mammograms and localizing lesions using only image-level annotations. Due to lesions in mammograms being very small relative to the entire image, a large number of non-tumor regions can seriously interfere with the classification performance. Therefore, the core idea of the algorithm is to identify regions that are highly likely to contain lesions in the mammogram and then calculate the overall probability that a mammogram is benign or malignant. Meanwhile, lesion regions play a key role in mammogram classification; clinical studies have suggested that both the local details (such as the shape of the lesion) and the overall structure (such as the density and pattern of breast fibroglandular tissue) are necessary for an accurate diagnosis [17,18]. Due to the high variability, intricate appearance, and sparsity of breast lesions, it is even difficult for professional radiologists to diagnose them [19,20]. To overcome this obstacle, we subdivide the mammogram classification into two tasks: candidate lesion region selection and classification. Shu et al. [21] have explored two pooling structures to score the mammogram regionally and select candidate lesion regions according to a threshold. The results show that the prediction of a convolutional neural network (CNN) depends on the activation value of each spatial position on its feature map, and the spatial position with a relatively large or small value can usually correspond to the visual position of the object. However, this method depends on the selection of the ratio of selected candidate lesions, but the size and number of lesions in different samples are different, so the performance of the model is limited due to these parameters. To address these issues, a local extremum mapping (LEM) method is designed in this study. In the proposed LEM, all regions are scored for generating a score map, and the local extremum positions in this map are recorded as candidate lesion regions. Different from selecting candidate regions by threshold, the regions corresponding to the local extremum positions are more representative than their surrounding regions. These most representative extremum scores are collected to calculate the benign/malignant probability of the mammogram, and the lesions are localized on the basis of these positions. To further enhance this process, during training, a sparse loss function is designed to stimulate the emergence of extreme values in the score map. In the stage of lesion localization, the extreme positions will be screened out and backpropagated, and the pixels that contribute the most to those extreme values are considered the lesion pixels. In this process, the local maximum points are defined as corresponding to the malignant area, while the local minimum points correspond to benign areas.

In general, the proposed framework can be summarized into three stages: extracting features with a deep convolutional network, calculating the local extremum map based on the features, and backpropagating response points to obtain the location of lesions. Compared to pixel- or object-level labels that need to be manually annotated, information on whether a mammographic image contains benign or malignant lesions can easily be obtained from the pathology diagnosis. For lesion locating, the proposed method expands the existing deep convolutional network so that it can filter suspicious regions in the mammogram and finally realize the functions of locating lesions. This structure can be embedded into any CNN as a supplementary module, with little computational overhead, and it does not change the original CNN architectures. In summary, the main contributions of this work include the following:

- A weakly supervised mammogram classification and localization model is proposed, which can locate lesions without detailed segmentation or detection annotations.
- A local extremum mapping method is proposed that utilizes the extrema as criteria to select candidate lesion regions. It is a more comprehensive way to extend the feature distance between benign and malignant lesions.
- Based on the above method, a sparse loss function is proposed to facilitate the generation of extremes and limit the number of extremes, and a localization algorithm is designed to obtain pixel-level lesion locations by backpropagating the extremes.

## 2. Related Work

After decades of development, deep neural networks have continuously made breakthroughs [22,23] and made great progress in computer vision [24,25,26]. For many big data analysis problems, deep learning models demonstrate significantly better performance than traditional methods [27,28]. Therefore, a multitude of methods to apply deep learning in medical image analysis have appeared [29,30,31]. Due to the rapid development of deep neural networks in medical image analysis, some deep learning methods have also been explored to solve the problem of automatic breast cancer screening. Most of these methods have demonstrated performance beyond traditional methods, and the models are easy to construct and more practical [11,12,32].

In mammographic image analysis, most DNN-based methods have two stages: segmentation or extraction of regions of interest (ROI), and ROI classification. Some works used DNN to classify breast masses in mammographic images [32,33,34]. Li Shen et al. [35] proposed a path-based method to detect and classify breast cancer lesions on mammograms. Al-antari et al. [36] constructed a complete framework for breast cancer detection, segmentation, and classification using digital X-ray mammograms. However, several recent studies have noted that these methodologies rely too much on ROI annotations and have poor scalability and repeatability. Chougrad et al. [37] collected lesion patches from mammographic images using segmentation annotations and built a lesion classification network to predict benign and malignant lesions. Agnes et al. [38] presented an end-to-end classification network implementing multilevel dilated convolutions to classify mammogram images. They further presented a deep ensemble transfer learning method and a deep classifier for feature extraction and mass classification [39]. The study in [40] addressed the problem of screening breast cancer in mammograms by converting it into a multiple instance learning (MIL) problem. They applied the sparsity assumption to the task of breast cancer classification to suppress the number of activated regions. Differently, the sparse loss in the proposed structure is intended to limit the non-extreme regions, make them fall within a range, and make the extreme values more distinguishable.

Deep weakly supervised localization methods with only image-level annotation as supervision typically aggregate deep responses to derive global confidence scores. Therefore, various pooling structures have been proposed to optimize the mapping of feature maps to classification probability. Global max pooling [41] selects the highest-response regions for category probability estimation, but it discards a significant amount of information. The global average pool [42] assigns equal importance to all regions, making it difficult to distinguish objects from the background. To address these limitations, Sun et al. [43] introduced log–sum–exponential pooling, which smoothly integrates the benefits of max and average pooling to focus on class-aware regions. Durand et al. [44] proposed rank max–min pooling, which selects high-scoring regions as positive and low-scoring ones as negative to improve feature discrimination. Zhou et al. [45] applied class peaks to image segmentation, showing that outliers in features are more informative. Shen et al. [14] proposed an attention-based CNN for the weakly supervised localization of malignant tumors in high-resolution mammographic images. Liang et al. [15] proposed a self-training strategy combined with CAM activation maps to replace attention to breast cancer localization. Sampaio et al. [16] explored the weakly supervised localization performance of mammography images using different activation map methods, such as CAM [42], GradCAM [46], and GradCAM++ [47].

However, these existing methods focus on extracting deep features from a global perspective while overlooking local spatial correlations, which are less suited to mammographic image analysis. Most mammogram lesions are small, and their classification depends not only on shape and size but also on their spatial distribution, which requires the consideration of local spatial relationships. For mammographic images, Han et al. [34] proposed a deep location soft-embedding-based network with regional scoring to evaluate and capture lesion location information, but they introduced an additional complex position embedding module for localization. Therefore, this paper designs a local extremum mapping mechanism to bridge feature space and image space. Specifically, the proposed local maximum mapping captures the spatial characteristics of suspected malignant lesions, while the local minimum mapping associates regions with benign lesions. By stimulating the emergence of these local extrema, the proposed method enables a comprehensive exploration of all regions of the image, enhancing the localization capability of the network. Furthermore, the proposed method can be simply integrated into any CNN architecture without modifying the original model structure, making it particularly beneficial for medical image analysis, where detailed annotations are time-consuming to obtain.

## 3. Method

In this section, a weakly supervised mammogram classification and localization technique is presented utilizing a local extremum map. CNN-based classifiers can produce the class activation map (CAM) [42] that specifies the contribution of each location in the image to the classification confidence. According to CNN’s feedforward process, it can be deduced that the abnormal point in the CAM usually corresponds with the visual cues of the target object, and in the mammogram, it is the lesion. Therefore, a mapping method based on the local extrema and the corresponding loss function is proposed to enhance the extremum in the CAM. In addition, considering that the lesion area in the mammographic image is small, a constraint term is added to the loss function to ensure the sparsity of the number of extrema. During the lesion localization phase, the extrema are backpropagated to generate maps that highlight informative regions for each object. The proposed mapping method retrieves all abnormalities in the mammogram, and the extrema are usually the most representative points in a local area, which can better locate the lesion.

### 3.1. The Overall Structure

**Figure 1:**
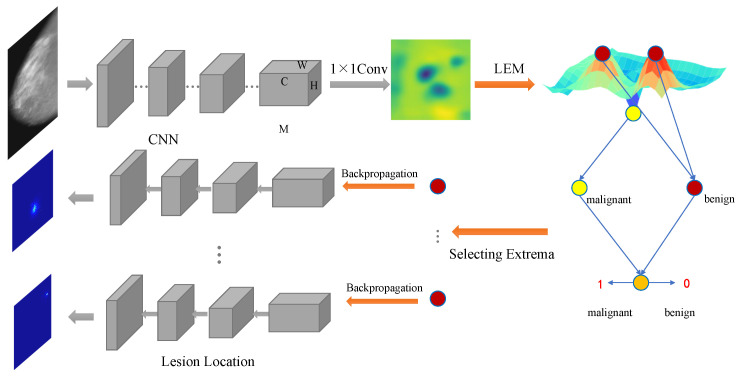
The overall architecture integrates the proposed LEM into a convolutional neural network for mammography image analysis. Feature maps extracted via the CNN are processed via the LEM, which computes the score map to find all local extrema. Based on these extrema, the classification confidence of the input mammography image is calculated, and the regions that tend to contain lesions are localized via backpropagation.

The dataset used in the proposed framework contains mammograms and image-level annotations. Given a mammogram, x, its corresponding label is *t*. When it is fed into a standard CNN-based structure, a feature map can be obtained from the last convolutional layer. Let $M\in R^{C\times W\times H}$ denote the feature map, where *W* and *H* represent the row and column size of the feature map, respectively, and *C* is the channel dimension. Most CNNs use global average pooling (GAP) [42] to obtain feature vectors from M, followed by a fully connected layer to compute the final classification confidence. By passing the parameters of this fully connected layer to a new convolutional layer with a kernel size of 1×1, it can be seamlessly converted into a structure with the ability to retain spatial information throughout forwarding. This process can be formulated as

(1) $$y^{gap}=\sigma \left[\sum_{c=1}^{C}\left(\frac{1}{WH}\sum_{i=1}^{W}\sum_{j=1}^{H}\left(M_{c,i,j}\right)\right)V_{c}\right]=\sigma \left[\frac{1}{WH}\sum_{i=1}^{W}\sum_{j=1}^{H}\sum_{c=1}^{C}\left(M_{c,i,j}V_{c}\right)\right],$$

where $y^{gap}$ denotes the category confidence calculated via GAP, $M_{c,i,j}$ is the element of row *c*, column *i*, and channel *j* in the tensor M, and V is the weight matrix of the fully connected layer or convolutional layer. Since mammogram classification is treated as a binary classification problem, and only one neuron is used to represent the predicted probability (as shown in Figure 1), the size of V is C×1 and its *c*-th element is denoted as $V_{c}$. The σ denotes the sigmoid active function that maps the confidence to the range of (0,1). According to Equation (1), the structure of a GAP followed by a fully connected layer can be implemented with a convolution layer of 1×1 followed by a GAP. The latter is used in this work to construct the network. So, a score map, $S={\left[S_{i,j}\right]}_{W\times H}$, can be obtained through this convolutional layer via

(2) $$S_{i,j}=\sum_{c=1}^{C}\left(M_{c,i,j}V_{c}\right),$$

where $S_{i,j}$ is the score of position (i,j) in the score map S. The values in S can well reflect the importance of all regions in the input image x, so the most informative extreme values in S will be scanned out for a further calculation of category confidence. Figure 1 shows the detailed structure of the proposed method.

### 3.2. Local Extremum Mapping

In order to drive the extrema in the score map to be more prominent, a local extremum mapping layer is added to follow the last convolution layer. There are two types of local extrema in the score map, the local maxima and the local minima, corresponding to the suspected malignant and suspected benign lesions (masses or calcification), respectively. During the feedforward calculation pass, two sampling maps, $S^{max}={\left[S_{i,j}^{max}\right]}_{W\times H}$ and $S^{min}={\left[S_{i,j}^{min}\right]}_{W\times H}$, are generated to locate all the maximum and minimum positions, which will be aggregated to compute the benign or malignant tendency of the lesion. In both sampling maps, the element at position (i,j) is equal to 0 or 1 (1 indicates the positions of the extremum). They are formed as

(3) $$S_{i,j}^{max}=\left\{\begin{array}{cc} 0 & if\;S_{i,j}\neq max\left(S_{i,j}^{srd}\right),\\ 1 & if\;S_{i,j}=max\left(S_{i,j}^{srd}\right), \end{array}\right.$$

and

(4) $$S_{i,j}^{min}=\left\{\begin{array}{cc} 0 & if\;S_{i,j}\neq min\left(S_{i,j}^{srd}\right),\\ 1 & if\;S_{i,j}=min\left(S_{i,j}^{srd}\right), \end{array}\right.$$

where $S_{i,j}^{srd}$ is the point set located around the point at coordinate (i,j) in score map S. When it is on the corner of the score map (such as the top left corner $S_{1,1}$ and the bottom right corner $S_{W,H}$), there are 4 points around it. When it is on the boundary and not the corner (such as the left boundary $S_{j,H}$ for ∀j∈(1,W)) of the score map, there are 6 points around it. Otherwise, there are 9 points around it. They are used to query extremum points.

When all extrema are found, the maxima $S^{max}$ and minima $S^{min}$ will be aggregated separately to calculate the probability that those suspicious regions tend to be malignant or benign using

(5) $$\begin{array}{cc} & a^{m}=\sum_{i,j}\left(S_{i,j}^{max}\cdot max\left(S_{i,j},0\right)\right),\\ & a^{b}=\sum_{i,j}\left(S_{i,j}^{min}\cdot min\left(S_{i,j},0\right)\right),\\ & y^{lem}=\sigma \left(\frac{a^{m}+a^{b}}{\sum_{i,j}\left(S_{i,j}^{max}+S_{i,j}^{min}\right)}\right), \end{array}$$

where $a^{m}$ indicates the aggregate value at which the mammogram tends to be malignant, $a^{b}$ denotes the aggregate value at which the mammogram tends to be benign, and $y^{lem}$ indicates the final probability of malignancy. As Equation (5) shows, the calculation of probability only uses extremum values as input.

When Equations (1) and (5) are compared, it can be seen that all scores are densely sampled in the conventional method to compute the confidence of the category. When the imbalance between the lesion area and the background area is taken into account, the proposed method sparsely samples the score map and only selects regions with abnormal scores to calculate the probability of the category. In this way, it is possible to avoid the problem in which the feature distance between benign and malignant lesions is too close because of sampling too many background regions.

### 3.3. Sparse Loss Function

**Figure 2:**
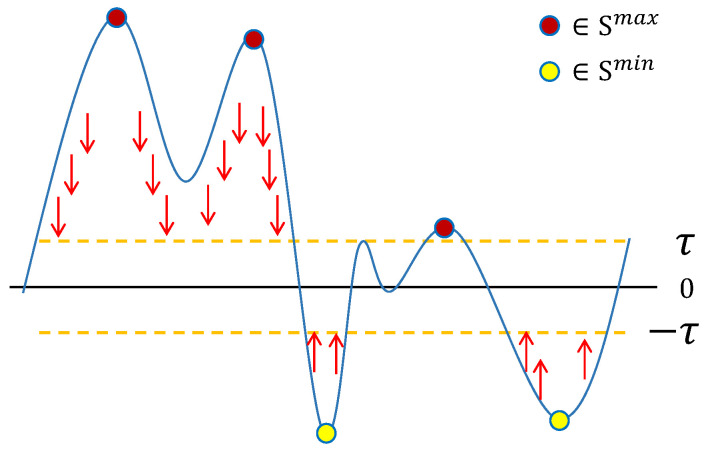
Schematic diagram of the gradient direction in the score map. The red and yellow points in the figure correspond to the maximum and minimum values, respectively. A sparse loss makes all non-extreme points outside the interval (−τ,τ) approach this range so as to make the extreme points more significant.

This section introduces the details of the sparse loss function designed for LEM. Mammography image classification is essentially a problem of classifying lesions. However, the lesion typically comprises only a tiny percentage in a mammogram, which means that most of the mammogram area contributes little to helping identify whether a mammogram is malignant or benign. In other words, the region of the lesion is relatively sparse throughout the mammogram. Correspondingly, when sampling scores, the active points in the score map should also be limited so that they are sparse. To constrain the number of active points and ensure that there is a small number of significant extrema, a sparse loss function $L_{spa}$ is first designed to constrain the LEM and defined as

(6) $$\begin{array}{cc} & L_{spa}=∥L_{eli}{∥}_{1}+\gamma {∥L_{pna}∥}_{1},\\ & L_{eli}=\sum_{i,j}\left[S_{i,j}^{max}\cdot \sigma \left(min(S_{i,j},0)\right)+S_{i,j}^{min}\cdot \sigma \left(-max(S_{i,j},0)\right)\right],\\ & L_{pna}=\sum_{i,j}\left[\sigma \left(max(S_{i,j},\tau )\right)+\sigma \left(-min(S_{i,j},-\tau )\right)\right], \end{array}$$

where $L_{eli}$ is the elimination loss used to eliminate the wrong extrema in the score map, and $L_{pna}$ is the penalty loss to ensure the activation values are sparse. And the parameter γ is a penalty factor, ${∥\cdot ∥}_{1}$ denotes the norm $L_{1}$, and the τ in $L_{pna}$ is a threshold used to control the sparsity range. The effect of this function is shown in Figure 2.

In addition, the LEM loss function, $L_{lem}$, and the GAP loss function, $L_{gap}$, are calculated to stimulate the appearance of extrema in the score map:

(7) $$\begin{array}{cc} & L_{gap}=g\left(y^{gap},t\right),\\ & L_{lem}=g\left(y^{lem},t\right),\\ & g(y,t)=t\cdot \log y+(1-t)\cdot \log(1-y). \end{array}$$

where t∈{0,1} is the image-level annotation (0 denotes benign, and 1 denotes malignant) for mammographic image x. The cross-entropy function *g* is used to evaluate the difference between the predicted value and the label.

The complete loss function is formulated as

(8) $$\begin{array}{c} J=\alpha (\mu ∥L_{spa}{∥}_{1}+L_{lem})+\left(1-\alpha \right)L_{gap}+\frac{\lambda }{2}{∥\theta ∥}^{2}, \end{array}$$

where θ is the parameter of the network, ∥·∥ denotes the $L_{2}$ norm, λ is the regularizer that controls model complexity, and μ is the sparsity factor, balancing the sparsity assumption with the importance of different regions. α∈[0,1] is the parameter used to dynamically control the choice of using GAP or LEM during the training phase. When α=0, the proposed method degenerates into using CNN with GAP to compute the final classification confidence and lesion localization, whereas, when α=1, only the proposed LEM is used. The motivation is that, at the beginning of the training stage, the generation of extrema is random, but candidate extrema can be generated by training with GAP. So, α is designed to control this process. During the training process, the gradient $\frac{\partial J}{\partial \theta }$ is computed based on the total loss defined in Equation (8):

(9) $$\begin{array}{c} \frac{\partial J}{\partial \theta }=\alpha \mu \frac{\partial \|L_{spa}{\|}_{1}}{\partial \theta }+\alpha \frac{\partial L_{lem}}{\partial \theta }+\left(1-\alpha \right)\frac{\partial L_{gap}}{\partial \theta }+\lambda \theta . \end{array}$$

**Figure 3:**
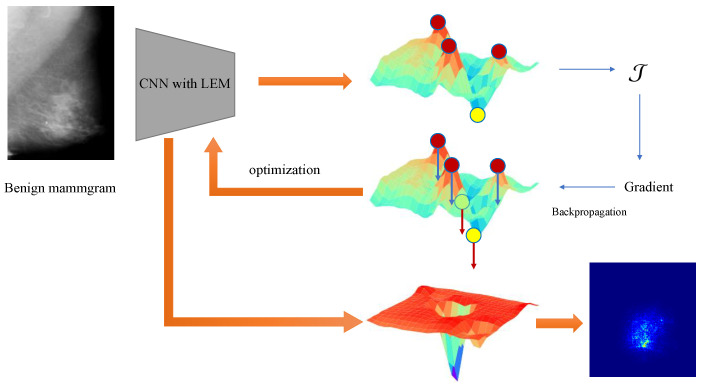
The training phase using the proposed sparse loss function for LEM. For benign mammography images, the gradient generated via this loss will make the local extremum in the score map that meets the condition of the loss function smaller. Finally, the local maximum values are suppressed, and the local minima become more prominent. In malignant samples, the direction of the gradient is the opposite.

By controlling the sparsity of the extrema and the training phase in this way, the adaptive potential of the network can be fully utilized. During classification, only a few regions are activated, which will make the activated regions more representative. This process is shown in Figure 3.

### 3.4. Lesion Localization

To locate the lesion, the contribution of each pixel in the mammogram to the confidence in the final output must be calculated. The CAM-based visualization structure (such as grad-cam, grad-cam++, etc.) calculates the importance of each region based on the last convolution layer, which has a good effect on big objects but is not fine-grained enough. Currently, most CNNs adopt ReLU as the activation function, leading to the fact that only weights greater than 0 contribute to the calculation. This conclusion has been proven in [48] and [45]. Therefore, for the purpose of calculating the importance of each pixel to a certain extremum, this property of CNN is utilized for reverse calculation in the proposed model. Unlike CAM, the final confidence in our model is not directly used to calculate the location map. All extrema will be searched, and those that meet the selection criteria will be backpropagated. Formulaically, the feature maps of two adjacent layers, *l* and l+1, are defined as $M^{l}$ and $M^{l+1}$, respectively. $n_{l}^{i}$ represents the *i*-th neuron in the layer *l*, and $n_{l+1}^{j}$ represents the *j*-th neuron in layer l+1. Therefore, $M_{i}^{l}$ and $M_{j}^{l+1}$ are the activation values of neurons $n_{i}^{l}$ and $n_{j}^{l+1}$, respectively.

In the feedforward calculation process, the activation value $M_{j}^{l+1}$ of the neuron $n_{j}^{l+1}$ is computed via $M_{j}^{l+1}=ReLU\left(\sum_{i\in C_{j}}U_{i,j}M_{i}^{l}+b_{l}\right)$, where $U_{i,j}\in \theta$ is the weight from neuron $n_{i}^{l}$ to neuron $n_{j}^{l+1}$, $b_{l}$ is the bias in layer *l*, and $C_{j}$ is the set of child connection nodes for neuron $n_{j}^{l+1}$. Then, the importance values $P(M_{i,j}^{l})$ of the previous layer can be computed top-down, starting from that of the output units, as follows:

(10) $$\begin{array}{cc} \; & P\left(M_{i}^{l}\right)=\sum_{j\in P_{i}}P\left(M_{i,j}^{l}\right)P\left(M_{j}^{l+1}\right),\\ & P\left(M_{i,j}^{l}\right)=ReLU\left(U_{i,j}\right)M_{i}^{l}Z_{j}, \end{array}$$

Here, $P_{i}$ is the set of parent connection nodes of the neuron $n_{i}^{l}$, weights less than 0 are discarded through the function ReLU, and the term $Z_{j}={\left(\sum_{i\in C_{j}}ReLU\left(U_{i,j}\right)M_{i}^{l}\right)}^{-1}$ is a normalization factor that ensures the sum of importance up to 1, that is, $\sum_{i\in C_{j}}P\left(M_{i,j}^{l}\right)=1$. The distribution of importance of the last feature map can be obtained directly from the score map S. Note that the score values in S are not calculated using the function ReLU. Therefore, in this layer, positive weights contribute to maximum values, and negative weights contribute to minimum values. The last layer can be calculated via

(11) $$\begin{array}{c} \begin{array}{cc} & P\left(M_{i,j,c}^{max}\right)=ReLU\left(V_{c}\right)M_{i,j,c}S_{i,j}^{max},\\ & P\left(M_{i,j,c}^{min}\right)=-ReLU\left(-V_{c}\right)M_{i,j,c}S_{i,j}^{min}. \end{array} \end{array}$$

Through the above formula, the importance of each pixel in the image to different extrema can be calculated. Since the importance map is a spatial display of the contribution of pixels to the prediction, the salient regions in the importance map can be considered lesion areas when benign and malignant labels are used as supervision. The algorithms for the LEM training process and the location of the lesion are specified in Algorithms 1 and 2, respectively.
**Algorithm 1** Training process of LEM**Require:**Input: Mammograms and image-level annotations.Learning rate: lr**Ensure:**The learned parameters: θ1:**for** epoch=1:max_epoch **do**2:    Extracting features for each input image3:    Computing the score map S via Equation (2)4:    Detecting extrema via Equations (3) and (4)5:    Computing the malignant probability $y^{lem}$ via Equation (5)6:    Computing the gradient of J with respect to the parameters θ via Equations (8) and (9)7:    Updating the parameters θ: θ = θ − $lr\cdot \frac{\partial J}{\partial \theta }$8:**end for**

**Algorithm 2** Lesion localization
**Require:**
Input: A mammogram, x, and its label, *t*.A trained network with parameter θ.
**Ensure:**
The lesion location map
1:Forward x to obtain the feature map M;2:Compute the extreme position map $S^{max}$ and $S^{min}$ via Equations (3) and (4)3:Compute the malignant probability $y^{lem}$ for mammogram x via Equation (5)4:**if** $y^{lem}>0$ **then**5:    **for** $S_{i,j}^{max}$ in $S^{max}$ **do**6:        Backpropagation for computing the location map7:    **end for**8:
**else**
9:    **for** $S_{i,j}^{min}$ in $S^{min}$ **do**10:        Backpropagation for computing the location map11:    **end for**12:
**end if**
13:Locating lesions in the location map


## 4. Experiments and Results

In this section, the details of the two datasets in the experiment are first presented. Then, the experimental setup is briefly introduced, including the evaluation strategy, the evaluation metric, and the implementation details. Lastly, the results of the experiment and the corresponding analysis are demonstrated.

### 4.1. Dataset

The structure proposed in this study was validated on two public data sets. Both datasets are scan data of mammograms stored in DICOM format. Their details and pre-processing steps are as follows.

**CBIS-DDSM** [49]: The CBIS-DDSM database (Curated Breast Imaging Subset of DDSM) consists of mammograms, segmentation annotations, lesion patches, and pathological diagnoses. This repository boasts a diverse array of 753 cases exhibiting calcifications and 891 cases displaying masses, a total of 3071 images. For all experiments conducted in this study, we only utilized image-level cancer labels for model training purposes.

**INbreast** [50]: The INbreast database consists of full-field digital mammograms and precise annotations, formatted similarly to modern computer vision datasets. The mammography images in this database contain a wide variety of lesions, such as masses, calcifications, asymmetries, and distortions. The INbreast database boasts a total of 115 cases, encompassing 410 images with BI-RADS annotations. In this study, we utilized BI-RADS annotations (BI-RADS ∈{4,5,6} as malignant).

The CBIS-DDSM database uses the standard split provided by [49], with 85% of the data allocated to training and 15% to testing. Following [21], the INbreast dataset was randomly split into mutually exclusive subsets for training (80%) and testing (20%) subsets. The preprocessing of raw mammograms was carried out according to the method in the previous work of this study [21], and the size of mammograms was adjusted to a standard size of 800×800. Original mammograms were augmented. All images were standardized to ensure that the value of the pixels was located at [0,1]. To reduce the impact of overfitting, in the training stage, several data augmentation techniques were applied to mammography images, including random horizontal flipping, adjustments to image contrast and saturation, rotation within the range of −30 to +30 degrees, and the addition of Gaussian noise.

### 4.2. Training Details

In the experiment, all CNN architectures utilized were built upon publicly available code provided by their respective authors, with only the last fully connected layer altered with our proposed LEM and no other structural modifications. The parameters of the convolutional layers were initialized on ImageNet. For a fair comparison, all experiments took an identical setup: the same update strategy, the same learning rate, and the same stopping condition. The initial learning rate of all models was $1\times 10^{-4}$. The λ in the loss function was set to a very small value, $1\times 10^{-5}$, to prevent the inability to generate extreme values; the τ in Equation (7) was set to the balanced value 3, and the α was initialized to 0.1 and gradually increased to 1 (gradual transition from GAP-dominated to LEM-dominated). The learning rate underwent a gradual decay process every eight epochs with a decay factor of 0.98. The parameters were updated in mini-batches, utilizing a batch size of 32. The classification threshold value was set to 0.5, and the training process terminated after the 400th epoch. The proposed method was evaluated based on classification accuracy (ACC) and the area under the receiver operating characteristic curve (AUC) for the mammogram classification task, as well as the dice similarity coefficient (DSC) and the recall for the localization task.

### 4.3. Results and Analysis

#### 4.3.1. Evaluation with Different Backbone Structures

The proposed LEM can be employed as an auxiliary layer in any CNN-based model. To verify its reliability and effectiveness with different CNN structures as the backbone, a set of control experiments was designed to compare the model performance under different conditions. Specifically, several popular models were trained and tested: the ResNet [51] series (Resnet-18, Resnet-50, and Resnet-101) and the DenseNet [24] series (DenseNet-121 and DenseNet-169). The parameters of LEM remain consistent across all models. In addition, the input sizes of different CNNs are inconsistent, but they do not affect the calculation of the score maps. Therefore, the input sizes of all networks in these experiments are set to the same size of 800×800. Table 1 shows the results of the LEM structure applied to different CNNs on the INbreast dataset.

It can be seen from the results that LEM has a significantly positive effect on all CNN-based networks listed, especially on the Resnet-18 structure. The reason may be that Resnet-18 is lighter and has fewer parameters than other networks, making its extracted features more primitive and containing more complex information. The GAP it uses struggles to extract effective lesion information from such feature maps, resulting in poor network performance. In contrast, LEM can optimize the extraction of spatial information and enhance the learning of abnormal regions in the training phase, making it easier to locate lesion regions in order to distinguish their benign and malignant nature. In addition, Resnet-18 with LEM outperforms the standard Resnet-50 and Resnet-101 models. It shows that LEM can fully exploit the potential of networks and improve their upper limit. Building models with better performance under the same scale of parameters in such a way can help the application of CNN-based CAD systems.

#### 4.3.2. Evaluation of Different Mapping Methods

The proposed approach includes the mapping of feature maps to score maps. To evaluate whether LEM works efficiently, several pooling methods are compared with Resnet-18 as the backbone. Among them, global average pooling (GAP) [42] and global max pooling (GMP) [41] are the most widely used structures. Region-based group-max pooling (RGP) [21] and global group-max pooling (GGP) [21] are threshold-based pooling methods that have achieved good performance in mammogram classification. The experimental results are shown in Table 2.

The comparison results of different pooling structures show that the LEM achieves very competitive performance, with accuracy similar to the previous work, GGP, and far better than other methods. The AUC of LEM far exceeds that of other pooling methods, indicating its better robustness. This is consistent with the previous discussion suggesting that the sparse lesion hypothesis can benefit from the learning ability of the deep network itself. The LEM employs a more flexible candidate region selection strategy and constrains the score map with a sparse loss to fully exploit the potential of the network. In contrast to GGP and RGP, it does not suffer from inter-sample variation (because the number of selected regions can be different in different samples).

#### 4.3.3. Comparisons with Other Methods

In recent years, some studies have attempted to apply several well-performing CNNs to the classification of mammography images. However, they did not take into account the characteristics of mammography, in which the classification of lesions is very different from the classification of typical objects in natural images, nor did they take full advantage of the potential of neural networks. The proposed method is completely automated and does not require pixel-level labeling or staged training. Image-level labels can also be directly derived from pathological results, facilitating the complete implementation of end-to-end training. The method was compared with other methods in mammography analysis, as shown in Table 3 and Table 4. These results are quoted from the original text.

The results show that the end-to-end trained LEM achieves superiority in the classification of whole mammograms. The features derived from the LEM structure are beneficial for training the deep network with mammograms as input. The DenseNet-169-based structure achieves a competitive result compared to the GMIC and MS + BRS models, proving the robustness of the proposed LEM structure. It shows the effectiveness of the features obtained via LEM in medical images. In addition, the accuracy in CBIS-DDSM is significantly lower than that in INbreast, possibly because of the different annotations. INbreast database only contains BI-RADS annotations, while CBIS-DDSM publishes benign/malignant annotations. In the domain of deep learning, annotations are critical, and the results are significantly impacted by them. Furthermore, the inconsistency of the data source devices in two datasets is also a crucial factor in determining the results.

#### 4.3.4. Evaluation of Localization Performance

To further validate the lesion localization performance of the LEM, we calculated the pixel-level accuracy on the test set of the INbreast dataset and compared it with other segmentation models. The results are shown in the following Table 5.

**Figure 4:**
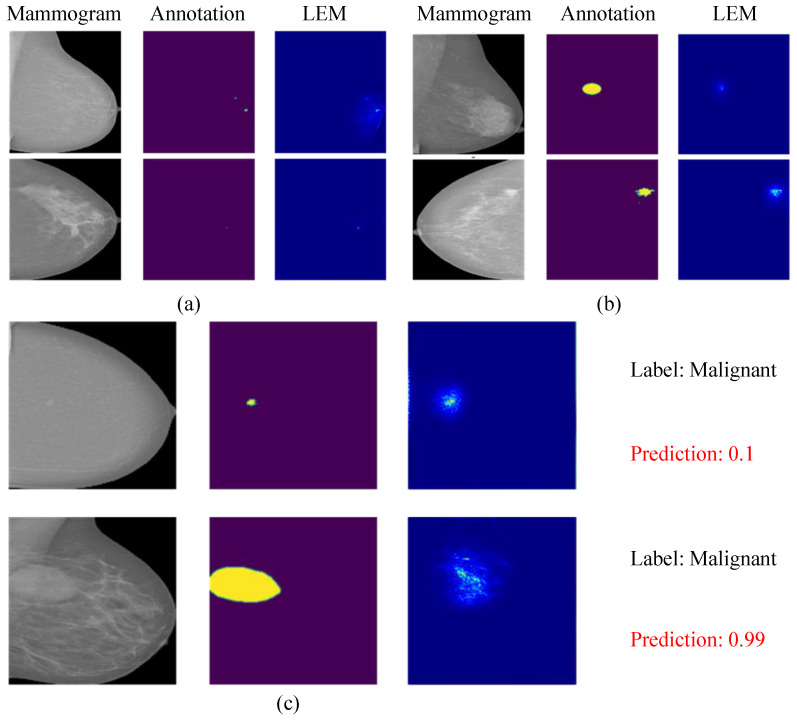
Visualization of whether sparse loss is used: (**a**) two samples containing calcification, (**b**) two samples with mass lesions, and (**c**) two samples that the model predicted incorrectly.

The results show that the locating performance of LEM is better than that of standard CNN without pixel-level annotation. This weakly supervised learning method can improve the positioning ability of the model. Figure 4a,b visualizes the positioning performance of the model for masses and calcifications. It can be found that the model will also pay attention to surrounding tissues when locating calcifications.

Figure 4c shows two cases of model prediction errors, namely, predicting benign lesions as malignant and predicting malignant lesions as benign. However, it can be seen from the location map that the lesion is detectable, although the model prediction is wrong. The reason for this phenomenon may be that the lesions have some of the same properties in features, such as shape and color. The model can distinguish the lesion from the background, but it cannot accurately classify some lesions with non-obvious features. These cases are also difficult to distinguish via the standard CNN, but the proposed model can provide the basis for the prediction as verification evidence for diagnosis.

#### 4.3.5. Ablation Study

In addition, we conducted ablation experiments to compare the performance of the model with and without sparse loss. The results are displayed in Table 6. As the results show, training the model with sparse loss increases the DSC by 12% but decreases the AUC by 0.3%. This indicates that the sparse loss does not have a positive effect on the classification performance of the model but can improve the localization performance of the model. The reason is that the sparse loss limits the number of extremes and enhances the association of extremes with lesions.

#### 4.3.6. Visualization of the Score Map

**Figure 5:**
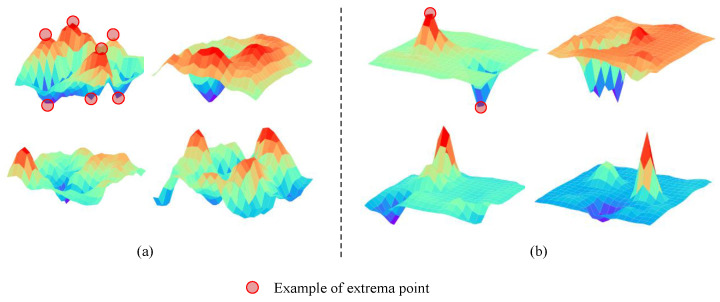
Visualization of lesion location map: (**a**) the samples visualized from the score map from LEM without sparse loss; (**b**) the LEM with sparse loss, resulting in fewer extrema.

In the comparative experiment on whether to use sparse loss, the visualization of the score map is shown in Figure 5. Figure 5a contains more extrema compared to Figure 5b, and the extremum values in Figure 5b are obviously higher than in Figure 5a. The network relies primarily on the most representative points. Therefore, training the model with sparse loss enhances the prominence of the lesion areas in the score map while effectively suppressing noise.

## 5. Conclusions

In this study, an end-to-end mammographic image diagnostic architecture has been proposed in which LEM was designed for mammogram classification and lesion localization without the need for pixel-level annotations. By identifying local extrema in the feature score maps, lesion-relevant regions were effectively captured, while non-discriminative areas were filtered out. Experimental results on the INbreast and CBIS-DDSM datasets demonstrated the effectiveness of the proposed LEM. However, there are still some limitations to lesion localization. The localization accuracy could still be improved, as small or subtle lesions may be underrepresented in the score maps. Future work will focus on enhancing feature refinement strategies and the integration of self-supervised or semi-supervised learning techniques to enhance location performance. In conclusion, the proposed method provides a practical solution for weakly supervised mammogram analysis, enhancing both classification accuracy and lesion localization performance while minimizing the reliance on costly pixel-level annotations. This is especially valuable in computer-aided diagnosis systems, for which reducing the cost of annotation is critical for scalability and efficiency.

## Figures and Tables

**Table 1:** Accuracy and AUC comparisons of different CNN with LEM on INbreast.

Method	ACC (%)	AUC
Resnet-18	87.7	0.890
Resnet-50	88.9	0.921
Resnet-101	87.7	0.940
DenseNet-121	93.8	0.953
DenseNet-169	88.9	0.942
LEM Resnet-18	92.6	0.951
LEM Resnet-50	92.6	0.953
LEM Resnet-101	95.1	0.946
LEM DenseNet-121	**96.3**	**0.976**
LEM DenseNet-169	91.4	0.957

Best results are highlighted in bold.

**Table 2:** Accuracy and AUC comparisons of different pooling structures applied to Resnet-18 on INbreast.

Methods	ACC (%)	AUC
GAP Resnet-18	87.7	0.89
GMP Resnet-18	85.2	0.841
RGP Resnet-18	91.9	0.930
GGP Resnet-18	91.4	0.936
LEM Resnet-18	**92.6**	**0.951**

Best results are highlighted in bold.

**Table 3:** Accuracy comparisons of the proposed framework and related models using the INbrest database.

Method	Pixel Label	ACC (%)	AUC
Random Forest [52]	Y	91.0	0.760
FrCN [53]	Y	95.6	0.948
Chougrad et al. [54]	Y	95.5	0.970
deep MIL [40]	N	90.0	0.89
FrCN [53]	N	91.1	0.906
RGP [21]	N	91.9	0.934
GGP [21]	N	92.2	0.924
DADA [55]	N	91.2	0.937
WMDNet [56]	N	94.7	0.936
CSAM [57]	N	94.9	0.961
WDCC [12]	N	93.4	0.943
DLSEN-RS [34]	N	95.1	0.936
MS+BRS [58]	N	96.3	0.971
LEM	N	**96.3**	**0.976**

Best results are highlighted in bold.

**Table 4:** Accuracy comparisons of the proposed framework and related models on CBIS-DDSM database.

Method	ACC (%)	AUC
PHResNet [59]	73.9	0.754
PA [60]	N/A	0.780
deep MIL [40]	74.2	0.791
RGP [21]	76.2	0.838
GGP [21]	**76.7**	0.823
MGBN [61]	74.5	0.825
GMIC [14]	N/A	0.840
WDCC [12]	77.0	0.834
DLSEN-RS [34]	74.2	0.753
LEM	76.5	**0.840**

Best results are highlighted in bold.

**Table 5:** DSC Comparisons of the proposed framework and UNet on INbreast database.

Method	Pixel Label	DSC	Recall
UNet [62]	Y	0.90	0.848
CAM [42]	N	0.25	0.49
grad-CAM [46]	N	0.29	0.52
LEM	N	0.37	0.56

**Table 6:** Comparisons of the sparse loss on INbreast database.

Method	DSC	AUC
without sparse loss	0.33	**0.979**
with sparse loss	**0.37**	0.976

Best results are highlighted in bold.

## Data Availability

The data presented in this study are openly available from CBIS-DDSM at https://www.cancerimagingarchive.net/collection/cbis-ddsm/, accessed on 14 September 2017 and INbreast at https://www.kaggle.com/datasets/ramanathansp20/inbreast-dataset, accessed on 1 May 2024.

## Abbreviations

The following abbreviations are used in this manuscript:

ACC	Accuracy
AUC	Area under the receiver operating characteristic curve
CAD	Computer-aided diagnosis
CAM	Class activation map
CNN	Convolutional neural network
DNN	Deep neural network
DSC	Dice similarity coefficient
GAP	Global average pooling
GGP	global group-max pooling
GMP	Global max pooling
LEM	Local extremum mapping
MIL	Multiple instance learning
RGP	Region-based group-max pooling
ROI	Regions of interest
